# Application of *Glycyrrhiza glabra* Root as a Novel Adsorbent in the Removal of Toluene Vapors: Equilibrium, Kinetic, and Thermodynamic Study

**DOI:** 10.1155/2013/986083

**Published:** 2013-03-11

**Authors:** Fazel Mohammadi-Moghadam, Mohammad Mehdi Amin, Mehdi Khiadani (Hajian), Fariborz Momenbeik, Heshmatollah Nourmoradi, Mohammad Sadegh Hatamipour

**Affiliations:** ^1^Environment Research Center, Isfahan University of Medical Sciences (IUMS), Isfahan, Iran; ^2^Department of Environmental Health Engineering, School of Health, IUMS, Isfahan, Iran; ^3^Department of Environmental Health Engineering, School of Health, Shahrekord University of Medical Sciences, Shahrekord, Iran; ^4^School of Engineering, Edith Cowan University, WA 6027, Australia; ^5^Department of Chemistry, Faculty of Sciences, University of Isfahan, Isfahan, Iran; ^6^Department of Environmental Health Engineering, School of Health, Ilam University of Medical Sciences, Ilam, Iran; ^7^Department of Chemical Engineering, Faculty of Engineering, University of Isfahan, Hezar Jerib Avenue, Isfahan 81746-73441, Iran

## Abstract

The aim of this paper is to investigate the removal of toluene from gaseous solution through *Glycyrrhiza glabra* root (GGR) as a waste material. The batch adsorption experiments were conducted at various conditions including contact time, adsorbate concentration, humidity, and temperature. The adsorption capacity was increased by raising the sorbent humidity up to 50 percent. The adsorption of toluene was also increased over contact time by 12 h when the sorbent was saturated. The pseudo-second-order kinetic model and Freundlich model fitted the adsorption data better than other kinetic and isotherm models, respectively. The Dubinin-Radushkevich (D-R) isotherm also showed that the sorption by GGR was physical in nature. The results of the thermodynamic analysis illustrated that the adsorption process is exothermic. GGR as a novel adsorbent has not previously been used for the adsorption of pollutants.

## 1. Introduction

Environmental pollution, as a result of industrial improvements, has created severe problems in the recent years. Volatile organic compounds (VOCs) are the major pollutants being released from various industrial plants and processes [1–4]. Some VOCs such as toluene existing in fuels, petroleum, and gasoline are widely used in many industrial solvents [5, 6]. Toluene vapors are released into the atmospheric environment over their production, transportation, application, and discharge every year [2]. Toluene can be absorbed by respiratory and gastrointestinal tracts. The human exposure to toluene results in neurotoxicity, mental depression, and various symptoms such as headache, fatigue and ataxia [7]. The US Environmental Protection Agency (USEPA) has considered toluene as a major pollutant, the concentration of which must be reduced to a very low level in the environment [8–10]. Various techniques including biofilter, biotrickling filter, bioscrubber, and adsorption have been successfully adopted to remove toluene vapors from gaseous media [1, 11–14].

Many adsorbents including zeolites [15–18], metal oxides [19], compost [11, 20], diatomaceous earth, chaff [20], ground tire rubber [11], and activated carbon [21–26] have been used for the removal of toluene vapors. Among the above-mentioned adsorbents, adsorption by activated carbon is one of the most common processes to remove VOCs, especially toluene, from gases solution, but, it is not cost effective [7]. *Glycyrrhiza glabra* is a herb that grows in various parts of the world as well as the southern areas of Iran [27]. It is a very sweet, humid, and soothing plant which has been used as a medicine to protect the liver, and to treat arthritis and the mouth ulcers for centuries in European and Eastern countries [27, 28]. The roots of *Glycyrrhiza glabra *are thick, long, cylindrical, fibrous, and multibranched [27] which after using its extract for medicine are disposed. In this study, the waste roots were, as a novel sorbent, used to remove toluene from gaseous solution. The main purpose of this study was to investigate the effect of various conditions including humidity, contact time, adsorbate concentration, and temperature on the adsorption of toluene vapor.

Literature review did not show any previous research using GGR waste as pollutant adsorbent. Only in a study, GGR was used as carbon sources in biological denitrification of drinking water [29].

## 2. Materials and Methods

### 2.1. Preparation of the Adsorbent

*Glycyrrhiza glabra* root (GGR) was provided by Rishmak Inc., an extract producing company located in Shiraz, Iran. The root of the herb was extracted at 3.5 bar pressure and 140°C temperature for 2 hours. Then dewatered GGR is dumped around the company as a waste material. In this paper, the GGR was ground to pieces of 0.5–1 cm, repeatedly washed with deionized water, dried in oven at 60°C for 48 h, and sterilized at 15 psi for 20 min.

### 2.2. Instrument

The surface area of GGR was determined with a multipoint N_2_ gas adsorption method (Sorptometer Kelvin 1042, Costech International, Italy). The chemical composition of the adsorbent was also characterized by X-ray fluorescence analyzer (Bruker, S4, Pioneer, Germany) and elemental combustion system (ECS 4010, Costech International, Italy). The concentration of toluene (purity of 99.5%, Merck, Germany) in the solution was quantified by a gas chromatography equipped with flame ionization detector (Agilent GC, 7890A, Netherland). The GC-FID procedure was optimized as follows.

The amount of 100 *μ*L of gaseous sample was injected into the instrument by 1 mL gastight syringes (Hamilton series no. 1001; Hamilton Co., NV, USA) equipped with Teflon Minnert fittings.

Helium (with flow rate of 1.11 mL/min) and H_2_ (with flow rate of 30 mL/min) were used as carrier gas and fuel gas, respectively. The characteristic of GC column was Agilent CP Sil 5: 30 m × 250 *μ*m × 0.25 *μ*m. The temperatures of the oven, injector, and detector were fixed at 100, 230, and 250°C, respectively.

The pH value and the particle size analyses of GGR were measured by digital pH meter and sieves with standard mesh, respectively. The bulk density and the water holding capacity analysis of adsorbent was conducted according to Ahn et al. [30].

### 2.3. Adsorption Experiments

The experiments including adsorbent humidity (0–70%), contact time (0–24 h), and adsorbate concentration (6.928 mg/L) were carried out at room temperature (25°C) in the 250 mL vials (with PTFE air-tight cap) and mixed by a rotary shaker (300 rpm for 24 h). The effect of temperature (10–50°C) on the adsorption was also determined as described above. After agitation period, 100 *μ*L of the polluted gas was analyzed for toluene by GC-FID. All the experiments were performed in triplicates and the mean values were considered. Blank samples were also employed to determine the amount of toluene loss. The blank recoveries ranged from 93.8 to 96% and the experimental data were adjusted for these recoveries. Calibration curve for determination of the toluene concentration was prepared according to the standard method [31].

The adsorbent capacity of GGR for toluene was calculated by

(1) $$\begin{array}{c} q_{e}=\frac{\left(C_{0}-C_{e}\right)V}{m}, \end{array}$$

where *q*_*e*_ (mg/g) is the adsorption capacity of GGR, *C*_0_ (mg/L) is the initial concentration of toluene, *C*_*e*_ (mg/L) is the equilibrium concentration of the toluene in the solution, *m* (g) is the mass of the adsorbent, and *V* (L) is the volume of the polluted gas (or volume of the vial).

## 3. Results and Discussion

### 3.1. Characterization of Media

The physical and chemical characteristics of GGR are presented in Table 1.

### 3.2. The Effect of Adsorbent Humidity

**Figure 1:**
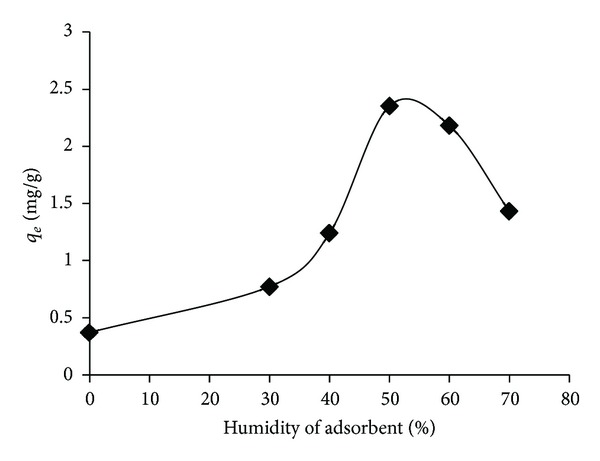
The effect of humidity of media on the sorption of toluene (adsorbent dose = 0.6 g, temperature = 25°C, contact time = 24 h, and adsorbate conc. = 6.928 mg/L).

The effect of adsorbent humidity (0–70%) on the sorption was determined. As can be seen in Figure 1, the adsorption capacity was expanded by increasing the sorbent humidity up to 50 percent. The sorbent humidity higher than 50% may be due to the occupation of the media porosity by water content, leading to the reduction of the adsorption capacity. Acuña et al. (2000) found out that the variation of water content of peat as an adsorbent for the toluene vapors did not have significant effects on the sorption rate [32]. GGR with a humidity of 50 percent was used for the subsequent experiments.

### 3.3. The Effect of Contact Time

**Figure 2:**
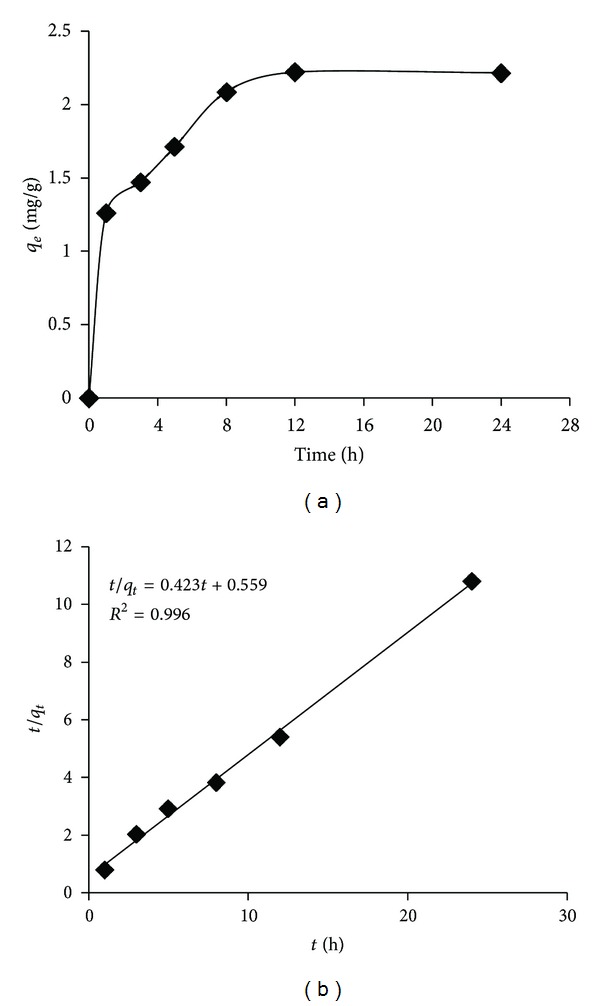
(a) The effect of contact time on the removal of toluene by GGR (adsorbent dose = 0.6 g, temperature = 25°C, adsorbent humidity = 50%, and adsorbate conc. = 6.928 mg/L). (b) Pseudo-second-order kinetic model.

Figure 2(a) shows the effect of contact time (0–24 h) on the adsorption of toluene by GGR. The adsorption as can be seen in the figure reached its maximum capacity by the elapse of time. 

The adsorption of toluene was quickly increased over the first hour of the sorption (1.3 mg/g) and steadily raised over the remaining contact time by 12 h since the adsorption sites are more accessible at the beginning of the sorption process and diminished by the passage of time. The contact time of 12 h was chosen for the rest of experiments. The adsorption capacity (*q*_*e*_) of GGR for the removal of toluene vapor was 2.2 mg/g over the 12 h contact time.  Table 2 compares the adsorption capacity of granular activated carbon (GAC), compost, diatomaceous earth, chaff, ground tire rubber (GTR), and GGR. The use of activated carbon may be prohibitive due to its high cost. The capacity of GGR in the removal of toluene is higher than the other natural adsorbents (e.g., compost, diatomaceous earth, and chaff).

Due to the suitable pH, water holding capacity, chemical composition (Table 1), and adsorption capacity, GGR is recommended as anew packing material for biofiltration. 

#### 3.3.1. The Adsorption Kinetics

Adsorption kinetic models can be helpful to specify the effectiveness of a sorbent in the removing pollutants and to determine the sorption mechanism type. The experimental data of toluene adsorption by GGR were analyzed via two common kinetic models including pseudo-first-order and pseudo-second-order models. The pseudo-first-order kinetic model is shown by

(2) $$\begin{array}{c} \ln\left(q_{e}-q_{t}\right)=\ln q_{e}-k_{1}t, \end{array}$$

where *q*_*e*_ (mg/g) and *q*_*t*_ (mg/g) are the quantity of toluene adsorbed onto GGR at equilibrium and at time (*t*), respectively. *k*_1_ (1/h) is the pseudo-first-order rate constant. *k*_1_ and *q*_*e*_ were calculated from the slope and intercept of the straight plotting ln⁡(*q*_*e*_ − *q*_*t*_) versus *t*, respectively [8].

The data obtained were also fitted by pseudo-second-order model. This adsorption kinetic can be correlated by [33]

(3) $$\begin{array}{c} \frac{t}{q_{t}}=\frac{1}{k_{2}q_{e}^{2}}+\frac{t}{q_{e}}.\; \end{array}$$

At the beginning stage of the adsorption, because *t* is nearly equal to zero, the initial adsorption rate, *h* (g/mg·h), could be represented by

(4) $$\begin{array}{c} h=k_{2}q_{e}^{2}, \end{array}$$

where *q*_*e*_ and *q*_*t*_ are the same as the pseudo-first-order model. *k*_2_ (g/mg·h) is the pseudo-second-order rate constant. *k*_2_ and *q*_*e*_ are obtained from the intercept and slope of *t*/*q*_*t*_ against *t* (see (3)), respectively [33].

The pseudo-second-order kinetic model for the removal of toluene vapor by GGR is presented by Figure 2(b). As shown, the higher correlation coefficient (*R* ^2^ = 0.996) acquired by this model with respect to the pseudo-first kinetic model (*R* ^2^ = 0.919) indicated that this model fitted the adsorption data better than the other kinetic models. Moreover, the calculated *q*_*e*,cal_ (2.36 mg/g) through this kinetic model is rationally closer to experimental *q*_*e*,exp⁡_ (2.22 mg/g) (Table 3).

### 3.4. The Effect of Adsorbate Concentration

**Figure 3:**
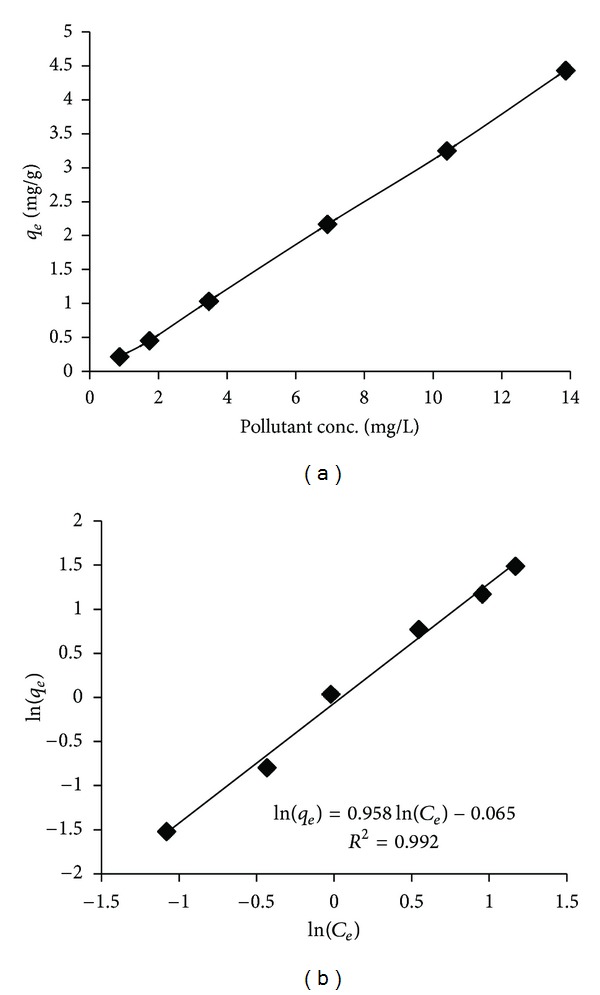
(a) The effect of toluene concentration on the adsorption by GGR (adsorbent dose = 0.6 g, temperature = 25°C, adsorbent humidity = 50%, and adsorbate Conc. = 0.86 to 13.86 mg/L). (b) Freundlich isotherm model.

The effect of initial toluene concentration in the range of 0.86 to 13.86 mg/L is presented in Figure 3(a). As it can be observed, the GGR is saturated more rapidly as the toluene concentration in the polluted air was increased. This may be due to rising of the driving force of toluene such as van der Waal's forces taking place at higher concentration of the pollutant.

#### 3.4.1. Adsorption Isotherms

Three adsorption isotherm models, Langmuir, Freundlich, and Dubinin-Radushkevich (D-R), were applied to analyze a relation between toluene concentration and the amounts of toluene adsorbed onto GGR.

The Langmuir isotherm forecasts the maximum monolayer adsorption capacity of the adsorbent [34]. The isotherm is shown by

(5) $$\begin{array}{c} \frac{C_{e}}{q_{e}}=\frac{C_{e}}{Q_{m}}+\frac{1}{bQ_{m}}, \end{array}$$

where *C*_*e*_ (mg/L) and *q*_*e*_ (mg/g) are the pollutant concentration and the adsorption capacity of the sorbent at equilibrium time, respectively. *b* (L/mg) is the Langmuir constant and *Q*_*m*_ (mg/g) is the maximum sorbent capacity. *Q*_*m*_ and *b* are calculated by the intercept and slope of the plot of *C*_*e*_/*q*_*e*_ against *C*_*e*_, respectively [34].

The Freundlich isotherm model was employed for multilayer adsorption on a nonuniform adsorbent surface [35]. This isotherm model can be described by

(6) $$\begin{array}{c} \ln q_{e}=\ln k_{f}+\frac{1}{n}\ln C_{e}, \end{array}$$

where *k*_*f*_ (L/g) and *n* are the isotherm constants. *k*_*f*_ and *n* are obtained from the intercept and slope of plotting ln⁡*q*_*e*_ versus ln⁡*C*_*e*_ (Figure 3(b)), respectively [35].

The isotherm parameters are presented in Table 4. The Freundlich isotherm model described the adsorption of toluene onto GGR very well (*R* ^2^ = 0.992). Oh et al. (2009) showed that the adsorption of toluene vapors by wet compost, and ground tire rubber was modeled well by Freundlich isotherm [11]. The strong bond between pollutant and adsorbent occurs when the *n* value obtained from the Freundlich isotherm is more than 1 [8]. Thus, the *n* value of 1.04 achieved by this isotherm model suggested that toluene vapor is properly adsorbed by the adsorbent. Singh et al. (2010) reported that *n* value, obtained from Freundlich isotherm of toluene vapor removal with wood charcoal, was 0.73 [1]. The strength of adsorption bond between adsorbate and adsorbent (*n* value) obtained by Singh et al. (2010) is weaker than that of our study. Oh et al. (2009) also revealed that the *n* value obtained for the sorption of toluene via wet compost and ground tire rubber was in the range of 0.96–1.13 [11].

The Dubinin-Radushkevich isotherm (D-R) specifies that the type of adsorption process is chemical or physical in nature [36]. The D-R isotherm can be described as

(7) $$\begin{array}{c} \ln q_{e}=\ln q_{m}-\beta \varepsilon^{2}, \end{array}$$

where *q*_*m*_ (mg/g) is the theoretical saturation sorption capacity, *β* (kJ/mol) is indicated as mean adsorption energy, and *ε* (Polanyi Potential) is equal to *RT*ln⁡(1 + 1/*C*_*e*_). *R* (kJ/mol·K) is the universal gas constant and *T* (K) is the temperature. *q*_*m*_ and *β* are determined by the intercept and the slope of plot of ln⁡*q*_*e*_ versus *ε* ^2^, respectively [36].

The type of adsorption process is specified by the *E* value. *E* (kJ/mol) is the mean adsorption energy that is given by

(8) $$\begin{array}{c} E=\frac{1}{\sqrt{2\beta }}\;. \end{array}$$

The *E* value in the range of 8–16 kJ/mol indicates that the chemical ion exchange occurs. *E* < 8 kJ/mol indicates physical and *E* > 16 kJ/mol chemical sorption process [34].

Table 4 shows that the *E* value of toluene adsorption by GGR is equal to 1.38 kJ/mol. Therefore, the adsorption of toluene by GGR was identified as physical in nature.

### 3.5. The Effect of Temperature

The effect of temperature in the range of 10–50°C on the adsorption of toluene vapor by GGR was investigated. Equations (9)–(12) were used for the determination of thermodynamic parameters such as enthalpy (Δ*H*), Gibbs-free energy (Δ*G*), and entropy (Δ*S*) [8]:

(9) $$\begin{array}{c} \Delta G=-RT\ln k,\;\;\; \end{array}$$

(10) $$\begin{array}{c} k=\frac{q_{e}}{C_{e}}, \end{array}$$

(11) $$\begin{array}{c} \Delta G=\Delta H-T\Delta S, \end{array}$$

(12) $$\begin{array}{c} \ln k=\frac{\Delta S}{R}-\frac{\Delta H}{RT}, \end{array}$$

where *k* is the equilibrium constant and *C*_*e*_, *q*_*e*_, and *R* are the same as defined before. The Δ*S* (J/mol·K) and Δ*H* (kJ/mol) of the toluene sorption were calculated from the intercept and slope of the straight plotting ln⁡*k* against 1/*T* (see (12)), respectively [8].

**Figure 4:**
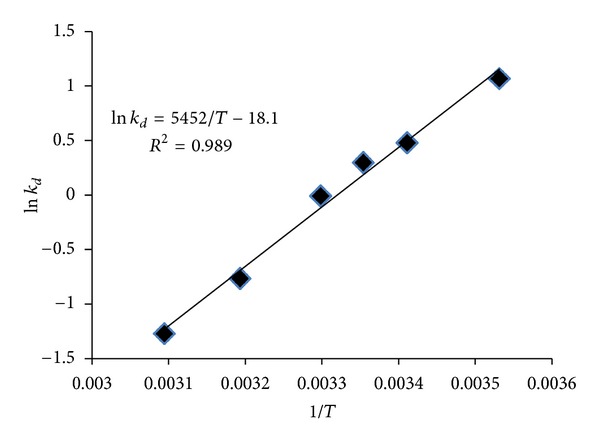
The effect of temperature of media on the sorption of toluene (adsorbent dose = 0.6 g, humidity= 50%, contact time = 12 h, and adsorbate conc. = 6.928 mg/L).

The effect of temperature on the adsorption is shown in Figure 4 and the thermodynamic parameters are presented in Table 5. According to Figure 4, the amount of toluene adsorption was reduced by increasing the temperature. The negative values of Δ*G* in the temperature range of 10–25°C (Table 5) suggested that the adsorption process was spontaneous and feasible, but its positive values indicated that the sorption was not favorable at the temperature of 30–50°C [33]. The reduction in the Δ*G* value by increasing temperature also indicated that the adsorption is not proper at higher temperatures [37]. Typically, the physical and chemical adsorption occurs while Δ*G* is between −20 to 0 kJ/mol and −40 to −400 kJ/mol, respectively [37]. The value of the Δ*G* obtained in this study demonstrated that the uptake of toluene by GGR is physical adsorption as it was found by D-R isotherm earlier. The negative value of adsorption enthalpy (Δ*H*) also supports that the adsorption process is exothermic in nature [37]. The comparisons between the toluene adsorption capacities at the various temperatures confirm this fact (Figure 4). The entropy change of toluene onto GGR was −150.48 J/mol·K. The negative value of Δ*S* indicated a reduction in the liberation of adsorbed toluene on GGR [38].

## 4. Conclusion

In this study, *Glycyrrhiza glabra *root (GGR) waste was used as a novel adsorbent for the adsorption of toluene vapor from gaseous media. The effect of different conditions including contact time, adsorbate concentration, humidity, and temperature on the adsorption was investigated. The pseudo-second-order kinetic model and Freundlich model fitted the adsorption data better than other kinetic and isotherm models, respectively. The D-R isotherm also showed that the sorption by GGR is physical in nature. The results of the thermodynamic analysis (negative value of obtained Δ*H*) corroborate that this adsorption process is exothermic. This adsorbent is a waste material with a sorption capacity of 2.2 mg/g. In comparison with other natural sorbents (e.g., compost, diatomaceous earth, and chaff), GGR seems to be a cost-effective sorbent in the removal of toluene vapor.

## Figures and Tables

**Table 1:** Characteristics of the GGR (on the dry basis).

Parameters	
Chemical composition (%)	

CaO	4.83
SiO_2_	1.76
Al_2_O_3_	0.552
Fe_2_O_3_	0.317
SO_3_	0.243
MgO	0.186
Na_2_O	0.072
K_2_O	0.068
P_2_O_5_	0.062
SrO	0.046
Cl	0.030
ZnO	0.019
TiO_2_	0.017
CuO	0.011
C	44.6
H	5.33
N	1.47

Physical characteristics	

pH	7.4
BET surface area (m^2^/g)	1.532
Particle diameter (cm)	0.5–1
Water holding capacity (g water/g dry GGR)	2.9
Bulk density (kg/m^3^)	37.62

**Table 2:** Comparison between adsorption capacities of different adsorbents.

Adsorbents	GAC [11]	diatomaceous earth [20]	Compost [20]	Chaff [20]	GTR [11]	GGR
Adsorption capacity (mg/g)	10.62	2.00	1.43	0.89	0.398	2.2

**Table 3:** Parameters of pseudo-second-order kinetic model obtained from this study.

Adsorbate	*q*_*e*,experimental_ (mg/g)	Pseudo-second-order model			
		*k*_2_ (g/mg·h)	*h* (g/mg·h)	*q*_*e*,calculated_ (mg/g)	*R* ^2^
Toluene	2.22	0.29	1.67	2.36	**0.996**

**Table 4:** Langmuir, Freundlich, and D-R isotherm parameters for the adsorption of toluene onto GGR.

Adsorbate	Langmuir isotherm			Freundlich isotherm			D-R isotherm		
	*Q*_*m*_ (mg/g)	*b* (L/mg)	*R* ^2^	*k*_*f*_	*n*	*R* ^2^	*q*_*m*_ (mg/g)	*E* (kJ/mol)	*R* ^2^
Toluene	3.76	0.18	0.724	0.94	1.04	**0.992**	3.14	1.38	0.887

**Table 5:** Thermodynamic parameters for the adsorption of toluene by GGR.

	Δ*G* (kJ/mol)						Δ*H* (kJ/mol)	Δ*S* (J/mol·K)
	283 K	293 K	298 K	303 K	313 K	323 K
Toluene	−2.72	−1.21	−0.46	0.29	1.80	3.30	−45.33	−150.48

## References

[1] Singh K, Singh RS, Rai BN, Upadhyay SN (2010). Biofiltration of toluene using wood charcoal as the biofilter media. *Bioresource Technology*.

[2] Mathur AK, Majumder CB, Chatterjee S (2007). Combined removal of BTEX in air stream by using mixture of sugar cane bagasse, compost and GAC as biofilter media. *Journal of Hazardous Materials*.

[3] Farhadian M, Vachelard C, Duchez D, Larroche C (2008). In situ bioremediation of monoaromatic pollutants in groundwater: a review. *Bioresource Technology*.

[4] Zamir SM, Halladj R, Nasernejad B (2011). Removal of toluene vapors using a fungal biofilter under intermittent loading. *Process Safety and Environmental Protection*.

[5] Su F, Lu C, Hu S (2010). Adsorption of benzene, toluene, ethylbenzene and p-xylene by NaOCl-oxidized carbon nanotubes. *Colloids and Surfaces A*.

[6] Bina B, Amin MM, Rashidi A, Pourzamani H (2012). Benzene and toluene removal by carbon nanotubes from aqueous solution. *Archives of Environmental Protection*.

[7] Tham YJ, Latif PA, Abdullah AM, Shamala-Devi A, Taufiq-Yap YH (2011). Performances of toluene removal by activated carbon derived from durian shell. *Bioresource Technology*.

[8] Nourmoradi H, Nikaeen M, Nejad MH (2012). Removal of benzene, toluene, ethylbenzene and xylene (BTEX) from aqueous solutions by montmorillonite modified with nonionic surfactant: equilibrium, kinetic and thermodynamic study. *Chemical Engineering Journal*.

[9] Yang C, Chen H, Zeng G, Yu G, Luo S (2010). Biomass accumulation and control strategies in gas biofiltration. *Biotechnology Advances*.

[10] Lin SH, Huang CY (1999). Adsorption of BTEX from aqueous solution by macroreticular resins. *Journal of Hazardous Materials*.

[11] Oh DI, Song JH, Hwang SJ, Kim JY (2009). Effects of adsorptive properties of biofilter packing materials on toluene removal. *Journal of Hazardous Materials*.

[12] Singh RS, Agnihotri SS, Upadhyay SN (2006). Removal of toluene vapour using agro-waste as biofilter media. *Bioresource Technology*.

[13] Pedersen AR, Arvin E (1995). Removal of toluene in waste gases using a biological trickling filter. *Biodegradation*.

[14] Standeker S, Novak Z, Knez Z (2009). Removal of BTEX vapours from waste gas streams using silica aerogels of different hydrophobicity. *Journal of Hazardous Materials*.

[15] Zhang W, Qu Z, Li X, Wang Y, Ma D, Wu J (2012). Comparison of dynamic adsorption/desorption characteristics of toluene on different porous materials. *Journal of Environmental Sciences*.

[16] Chen H, Zhang H, Yan Y (2012). Preparation and characterization of a novel gradient porous ZSM-5 zeolite membrane/PSSF composite and its application for toluene adsorption. *Chemical Engineering Journal*.

[17] Lee DG, Kim JH, Lee CH (2011). Adsorption and thermal regeneration of acetone and toluene vapors in dealuminated Y-zeolite bed. *Separation and Purification Technology*.

[18] Slioor RI, Kanervo JM, Keskitalo TJ, Krause AOI (2008). Gas phase adsorption and desorption kinetics of toluene on Ni/*γ*-Al_2_O_3_. *Applied Catalysis A*.

[19] Agelakopoulou T, Roubani-Kalantzopoulou F (2009). Chromatographic analysis of adsorption: chemisorption and/or physisorption. *Chromatographia*.

[20] Mudliar S, Giri B, Padoley K (2010). Bioreactors for treatment of VOCs and odours—a review. *Journal of Environmental Management*.

[21] Lillo-Ródenas MA, Fletcher AJ, Thomas KM, Cazorla-Amorós D, Linares-Solano A (2006). Competitive adsorption of a benzene-toluene mixture on activated carbons at low concentration. *Carbon*.

[22] Pei J, Zhang JS (2012). Determination of adsorption isotherm and diffusion coefficient of toluene on activated carbon at low concentrations. *Building and Environment*.

[23] Kim KD, Park EJ, Seo HO, Jeong MG, Kim YD, Lim DC (2012). Effect of thin hydrophobic films for toluene adsorption and desorption behavior on activated carbon fiber under dry and humid conditions. *Chemical Engineering Journal*.

[24] Takeuchi M, Hidaka M, Anpo M (2012). Efficient removal of toluene and benzene in gas phase by the TiO_2_/Y-zeolite hybrid photocatalyst. *Journal of Hazardous Materials*.

[25] San JY, Hsu YC, Wu LJ (1998). Adsorption of toluene on activated carbon in a packed bed. *International Journal of Heat and Mass Transfer*.

[26] Rochereau A, Marc B, Laurence LC, Mauret E, Albert S, Pierre LC (2008). Combined air treatment: effect of composition of fibrous filters on toluene adsorption and particle filtration efficiency. *Chemical Engineering Research and Design*.

[27] Gupta VK, Fatima A, Faridi U (2008). Antimicrobial potential of *Glycyrrhiza glabra* roots. *Journal of Ethnopharmacology*.

[28] Visavadiya NP, Narasimhacharya AVRL (2006). Hypocholesterolaemic and antioxidant effects of *Glycyrrhiza glabra* (Linn) in rats. *Molecular Nutrition & Food Research*.

[29] Ovez B, Ozgen S, Yuksel M (2006). Biological denitrification in drinking water using *Glycyrrhiza glabra* and *Arunda donax* as the carbon source. *Process Biochemistry*.

[30] Ahn HK, Richard TL, Glanville TD (2008). Laboratory determination of compost physical parameters for modeling of airflow characteristics. *Waste Management*.

[31] Lodge JPA (1988). *Methods of Air Sampling and Analysis*.

[32] Acuña ME, Pérez F, Auria R, Revah S (2000). Microbiological and kinetic aspects of a biofilter for the removal of toluene from waste gases. *Biotechnology and Bioengineering*.

[33] Senturk HB, Ozdes D, Gundogdu A, Duran C, Soylak M (2009). Removal of phenol from aqueous solutions by adsorption onto organomodified Tirebolu bentonite: equilibrium, kinetic and thermodynamic study. *Journal of Hazardous Materials*.

[34] Faghihian H, Nourmoradi H, Shokouhi M (2012). Performance of silica aerogels modified with amino functional groups in PB, (II) and CD, (II) removal from aqueous solutions. *Polish Journal of Chemical Technology*.

[35] Koyuncu H, Yıldız N, Salgın U, Köroğlu F, Çalımlı A (2011). Adsorption of *o*-, *m*-and *p*-nitrophenols onto organically modified bentonites. *Journal of Hazardous Materials*.

[36] Kul AR, Koyuncu H (2010). Adsorption of Pb(II) ions from aqueous solution by native and activated bentonite: kinetic, equilibrium and thermodynamic study. *Journal of Hazardous Materials*.

[37] Su J, Lin HF, Wang QP, Xie ZM, Chen ZL (2011). Adsorption of phenol from aqueous solutions by organomontmorillonite. *Desalination*.

[38] Nourmoradi H, Khiadani M, Nikaeen M (2013). Multi-component adsorption of benzene, toluene, ethylbenzene, and xylene from aqueous solutions by montmorillonite modified with tetradecyl trimethyl ammonium bromide. *Journal of Chemistry*.

